# The effect of animal versus plant protein on muscle mass, muscle strength, physical performance and sarcopenia in adults: protocol for a systematic review

**DOI:** 10.1186/s13643-022-01951-2

**Published:** 2022-04-13

**Authors:** Rachel J. Reid-McCann, Sarah F. Brennan, Michelle C. McKinley, Claire T. McEvoy

**Affiliations:** Centre for Public Health, Queen’s University Belfast, Institute of Clinical Science A, 1st Floor, Grosvenor Road, Belfast, BT12 6BJ UK

**Keywords:** Animal protein, Plant protein, Sarcopenia, Muscle mass, Muscle strength, Physical performance, Systematic review

## Abstract

**Background:**

The evidence base for the role of dietary protein in maintaining good muscle health in older age is strong; however, the importance of protein source remains unclear. Plant proteins are generally of lower quality, with a less favourable amino acid profile and reduced bioavailability; therefore, it is possible that their therapeutic effects may be less than that of higher quality animal proteins. This review aims to evaluate the effectiveness of plant and animal protein interventions on muscle health outcomes.

**Methods:**

A robust search strategy was developed to include terms relating to dietary protein with a focus on protein source, for example dairy, meat and soy. These were linked to terms related to muscle health outcomes, for example mass, strength, performance and sarcopenia. Five databases will be searched: MEDLINE, Scopus, Cochrane Central Register of Controlled Trials, Embase and Web of Science. Studies included will be randomised controlled trials with an adult population (≥ 18) living in the community or residential homes for older adults, and only English language articles will be included. Two independent reviewers will assess eligibility of individual studies. The internal validity of included studies will be assessed using Cochrane Risk of Bias 2.0 tool. Results will be synthesised in narrative format. Where applicable, standardised mean differences (SMD) (95% confidence interval [CI]) will be combined using a random-effects meta-analysis, and tests of homogeneity of variance will be calculated.

**Discussion:**

Dietary guidelines recommend a change towards a plant-based diet that is more sustainable for health and for the environment; however, reduction of animal-based foods may impact protein quality in the diet. High-quality protein is important for maintenance of muscle health in older age; therefore, there is a need to understand whether replacement of animal protein with plant protein will make a significant difference in terms of muscle health outcomes. Findings from this review will be informative for sustainable nutritional guidelines, particularly for older adults and for those following vegan or vegetarian diets.

**Systematic review registration:**

PROSPERO CRD420201886582

## Background

Sarcopenia is a debilitating condition that is characterised by loss of muscle mass and strength and is associated with a range of other health outcomes including reduced physical functional performance, weakness, frailty, falls, hospitalisation and death [1, 2]. It has been estimated that 30% of over 60s and 50% of over 80s have sarcopenia [3]. With the over 85s population in the UK expected to double in the next three decades, sarcopenia will be a greater public health concern than ever before [4].

An inadequate protein intake is a core modifiable risk factor for sarcopenia due to the role of dietary protein in supplying essential amino acids for muscle protein synthesis and therefore maintenance of muscle mass [3, 5]. Numerous longitudinal studies have indicated that a higher dietary protein intake is protective of muscle mass, strength and physical performance [6–9]. Likewise, there is good experimental evidence that protein supplementation is effective in improving muscle mass and strength in sarcopenic populations [10]. It is for these reasons that protein supplementation, alongside resistance training, is currently the standard treatment for sarcopenic patients [11]. Encouragement of adequate dietary protein intake as part of a healthy diet is also an important preventive measure. What is considered to be an adequate dietary protein intake for older adults is likely to be higher than that of younger populations due to age-related anabolic resistance of muscle protein synthesis [12]. For this reason, expert consensus suggests that older adults should consume an additional 0.2–0.7 g of dietary protein per kg body weight than younger adults daily in order to protect against muscle atrophy [13].

While the evidence base for the role of dietary protein in maintaining good muscle health in older age is strong, the importance of protein source remains unclear. There is evidence that equal amounts of protein from different sources are not met with an equal postprandial response in terms of amino acid absorption and metabolic utilisation. For example, modelling studies have found that soy protein experiences greater splanchnic extraction and nitrogen losses compared to milk protein [14, 15]. This is especially pertinent for older adults, given the increase in splanchnic extraction of amino acids associated with ageing and therefore the reduced free amino acid pool available for muscle protein synthesis [16]. These age-related changes in protein digestion combined with the varied postprandial response to different protein sources indicate that there may be important differences for the anabolic potential of different protein sources between younger and older adults.

There may also be important differences between male and female populations in terms of their anabolic response to different dietary proteins. There is evidence of sex dimorphism in protein metabolism and muscle protein synthesis, which is particularly evident during periods of life in which significant hormonal changes take place, e.g. menopause [17]. This suggests that the choice of protein source for conservation of muscle health will be particularly important in older age especially as later life is an important period of hormonal change for men and women alike.

**Fig. 1:**
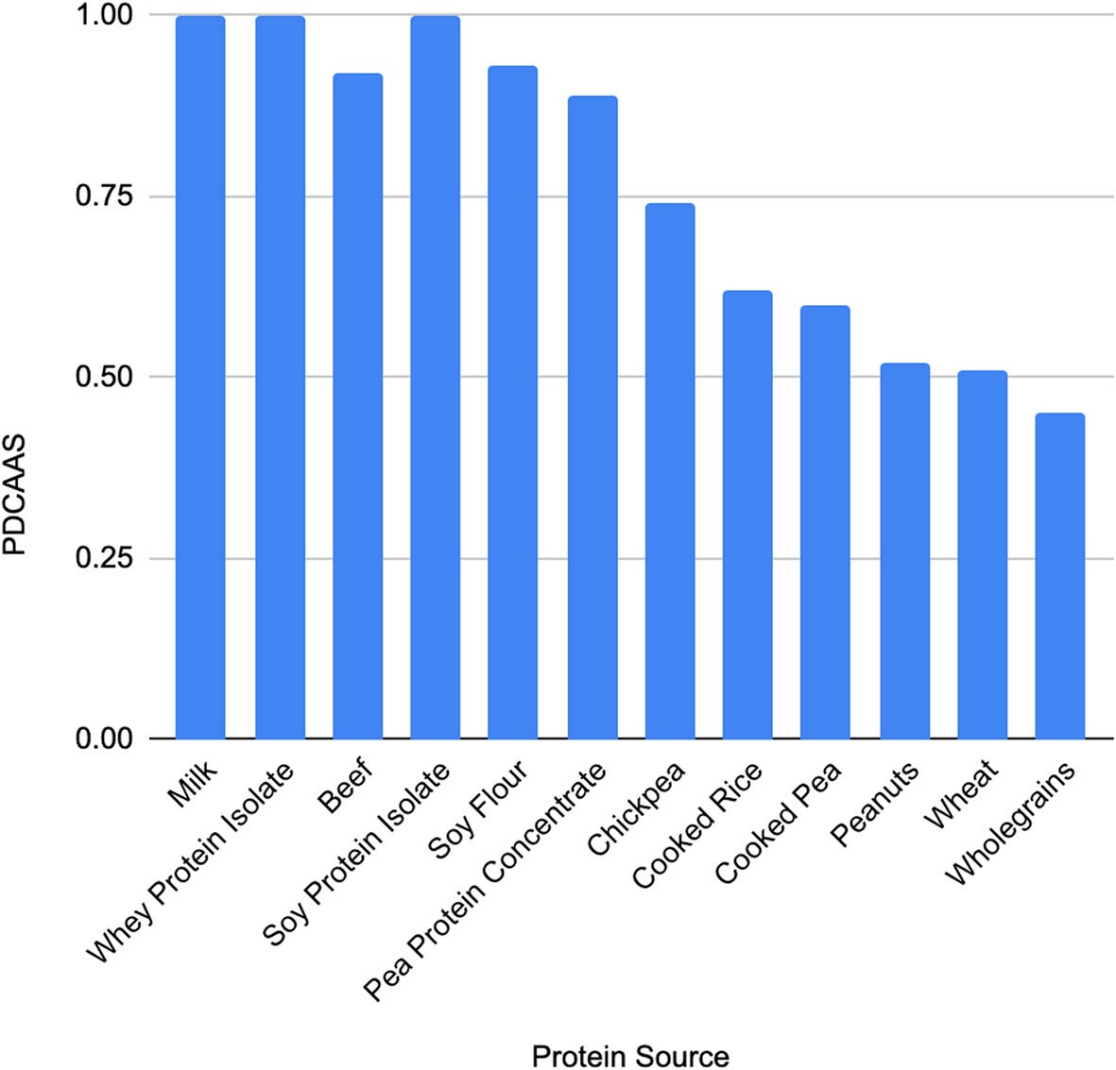
Protein digestibility-corrected amino acid score (PDCAAS) for 12 protein sources (source: Berrazaga et al. [19])

Proteins also inherently differ in their quality, i.e. their amino acid profile combined with their bioavailability. Proteins from animal food sources are referred to as high-quality proteins due to the presence of all nine essential amino acids (EAA) in high quantities as well as the greater bioavailability of these EAA. In comparison, plant proteins often have very little of one or several of the EAAs, for example many legumes lack methionine, cysteine and tryptophan [18]. They are also less bioavailable due to the structure of plant proteins and high concentration of compounds that bind protein, for example tannins and phytic acid [19]. A greater proportion of dietary fibre in plant protein food matrices is also expected to reduce protein digestibility [20]. Protein quality can be summarised using the protein digestibility-corrected amino acid score (PDCAAS) [21]. See Fig. 1 for an overview of PDCAAS for different protein sources.

Animal protein sources such as meat, fish and dairy have a consistently high protein quality, whereas the quality of plant protein sources is more variable (Fig. 1). This suggests that animal sources will be more effective for preserving muscle health during ageing. However, the encouragement of a greater consumption of animal protein sources for healthy muscle ageing may not be appropriate for optimising all outcomes related to diet. Animal products such as dairy are nutritionally rich, important dietary sources of calcium and protective of musculoskeletal health [22]. However, on the other hand, a plant-based diet has repeatedly shown to be associated with improved cardiovascular health outcomes and all-cause mortality [23, 24]. The optimum proportion of plant to animal food items in the diet in terms of optimising health outcomes is not currently known, and consideration must include the environmental impact of any recommendation to increase animal protein intake. The EAT-Lancet Commission, “Our Food in the Anthropocene: Healthy Diets from Sustainable Food Systems”, aims to develop global scientific targets based on evidence available for healthy diets and sustainable food production in order to meet the UN Sustainable Development Goals (SDGs) and Paris Agreement [25]. The lack of scientific targets to date is thought to have hindered efforts to transform the global food system, and it has been stated that current targets for carbon emissions will not be met if the current Westernised dietary pattern does not change in favour of a more plant-based diet [25].

Previous systematic reviews have attempted to distinguish the effects of different protein sources on muscle health outcomes including muscle mass and strength (Appendix 3). However, to our knowledge, previous reviews have not extended the scope to include important physical performance or sarcopenia outcomes for the ageing muscle [26–28]. Furthermore, reviews have been limited either by the sole inclusion of younger adults (< 40 years) [26] or by focusing primarily on soy plant proteins [27] rather than the comprehensive range of plant proteins that have been studied. Previous reviews also did not consider the effects of sex in analyses, yet there may be important sex differences in the impact of different protein sources on muscle health. Furthermore, energy deficit can impair muscle protein synthesis [29]; however, previous meta-analyses did not conduct separate subgroup analyses for the pooled effects of protein interventions with and without energy deficit [28].

This protocol for a systematic review outlines methodology that aims to add to the current knowledge base by introducing novel factors to address the aforementioned gaps: a wider scope in terms of muscle health outcomes, a comparison of effects by sex and independent statistical analyses of studies featuring energy deficits in the intervention.

### Hypotheses and research questions

We hypothesise that a similar weight of high quality, plant protein isolate (i.e. soy) is as effective as animal protein isolate (e.g. whey) for preserving muscle health during ageing. We hypothesise that interventions substituting whole animal protein foods (e.g. red meat) with plant proteins (e.g. soybeans) or whole plant diets (e.g. vegan diets) are not as effective owing to a potentially lower ratio of protein in plant protein foods.

The primary research question for this review is as follows:

- What is the effect of animal versus plant protein on muscle mass, muscle strength, physical performance and sarcopenia in adults?

Secondary research questions are as follows:

- Does the effect of animal versus plant proteins on muscle health differ between males and females?
- Does the effect of animal versus plant proteins on muscle health vary at different life stages (e.g. younger or older than 60)?
- How does the effect of different plant proteins (e.g. soy, wheat) compare to animal proteins for muscle health?

## Methods and design

The methods for this systematic review have been developed according to the recommendations from the Preferred Reporting Items for Systematic Review and Meta-Analysis Protocols (PRISMA-P) 2015 statement [30]. The protocol has been registered with PROSPERO: CRD42020188658.

### Inclusion criteria

#### Participants

Adults over the age of 18 are eligible for inclusion if they are either living in the community or in residential care homes for older adults. Hospitalised populations are excluded. Those with a disease that affects the normal absorption, metabolism or requirements of dietary protein are excluded, for example patients with cancer, chronic kidney disease or malnutrition (see Table 1).

**Table 1:** Inclusion and exclusion criteria for study screening

Variable	Criteria	Include	Exclude
Language	English	X	
Study design	Randomised controlled trial	X	
	Cohort studies, acute/mechanistic studies, reviews, protocols, conference abstracts		X
Population	Adults aged 18+	X	
	Children; pregnant women		X
Setting	Community; care homes for older adults	X	
	Hospitalised patients		X
Duration	≥ 4 weeks	X	
Intervention	Plant protein supplement or diet	X	
Comparator	Animal protein supplement or diet (similar protein and energy content to intervention)	X	
Outcome(s)	Muscle mass, muscle strength, physical performance, sarcopenia. Measured by one of the listed methods in Table 2	X	
Comorbidities	Non-alcoholic fatty liver disease; diabetes mellitus	X	
	Chronic kidney disease, cancer, malnutrition, Crohn’s disease, rheumatoid arthritis, fibromyalgia, HIV		X

#### Intervention(s)

The intervention in included studies is consumption of plant protein. This may be presented in various forms:

- Supplementation of diet with a whole food source of protein, e.g. tofu or beans
- Supplementation of diet with an isolated or concentrated form of plant protein, e.g. soy protein isolate powder
- A whole diet intervention in which protein sources are predominantly from plant sources, e.g. a vegan diet or a plant-based diet low in animal source foods

The intervention should have a minimum duration of 4 weeks as this time period has been shown to be sufficient for measurable hypertrophy to take place when combined with resistance training [33]. Studies that include such physical activity components can be included if the intervention and comparator follow the same training programme. Likewise, studies that provide micronutrients alongside both plant and animal interventions can be included provided these are identical, i.e. vitamin D supplementation in both arms.

#### Comparator(s)

The comparator will be a parallel intervention of animal protein. The comparator and intervention will have similar quantities or protein content in order for treatments to be comparable. As with the intervention, the comparator may be supplementation with a single animal protein source, for example isolated whey protein powder or a whole food such as a portion of chicken. Similarly, an animal-based/omnivorous diet may be compared to a diet based on plant protein sources given the quantities are comparable.

#### Outcomes

The outcomes of interest are mean change in muscle mass, muscle strength, physical performance and sarcopenia from baseline. These may be measured by a range of methods as listed in Table 2. Each outcome is equal in importance, and no additional outcomes are under investigation. The rationale for outcome choices is that muscle mass, strength and physical performance are altogether important determinants of sarcopenia and are each a component of the sarcopenia case definition.

**Table 2:** Appropriate methods of measurement for four outcome measures of interest in review

Outcome	Method of measurement
Muscle mass	Magnetic resonance imaging (MRI), computed tomography (CT), dual-energy X-ray absorptiometry (DEXA), bioelectrical impedance (BIA), hydrostatic weighing, air displacement plethysmography, appropriate anthropometric measures
Muscle strength	Appendicular skeletal muscle strength measured by, e.g. pinch strength, grip strength, one repetition maximum with free weights or resistance machines, any other acceptable isometric or dynamic strength tests
Physical performance	Timed-Up-and-Go (TUG) speed test, gait speed test, balance tests, short-performance physical battery (SPPB) test, repeated chair stands, any other functional test used in young or older adults to measure ability of muscle to perform a physical task
Sarcopenia	Using methods and cutoff points advised by the European Working Group on Sarcopenia in Older People (EWGSOP)^a^ or Asian Working Group for Sarcopenia (AWGS)^b^

^a^ [31]

^b^ [32]

#### Report characteristics

This review will include randomised controlled trials (RCTs) published in the English language before July 2020. Only full papers will be considered; conference abstracts are excluded as extraction of sufficient data and quality assessment may not be possible from the limited information given (Table 1).

#### Information sources and search strategy

An initial scoping review was conducted on MEDLINE using key search terms such as ‘dietary protein’ and ‘muscle’. This scoping exercise identified a sufficient number of randomised trials focusing on plant versus animal effects on muscle outcomes, particularly for optimising sports performance, for measuring effects of soy on menopause symptoms or as a part of a weight loss intervention. Relevant words or terms used in the titles and abstracts of these papers were identified and contributed to construction of a comprehensive search algorithm with the guidance of an information specialist. The final search algorithm is a combination of the reviewer’s own terms combined with standardised medical subject headings (MeSH). Two examples of this search strategy, tailored to the CENTRAL and Scopus databases, can be seen in Appendices 1 and 2.

Five databases will be searched in total: MEDLINE, Scopus, Embase, Web of Science and Cochrane Central Register of Controlled Trials (CENTRAL). A manual search of reference lists and recently published papers will be undertaken prior to data extraction to ensure any relevant papers not captured by the search will be included. Study authors will be contacted in any case of unclear or missing data.

### Study records

#### Screening and selection

Once searches are complete, all references will be downloaded to Endnote [version X9 3.2, Clarivate Analytics, PA, USA] and duplicates removed. Following this, studies will be uploaded to Rayyan [Qatar Computing Research Institute, Doha, Qatar] where titles and abstracts will be screened. Two reviewers (RRM, SB) will screen abstracts against inclusion criteria seen in Table 1 while blinded to each other’s decisions, and conflicts will be resolved through discussion between the other members of the review team (CME, MMK). Studies that meet the inclusion criteria at this stage will subsequently undergo blinded full-text screening by two reviewers (RRM, SB) using Rayyan. A PRISMA flow diagram will be developed to show the progress from the initial search to final selection of studies to be included in review.

#### Data extraction

A predefined template will be used for data extraction. A summary of variables to be extracted from each included study is provided in Table 3. One reviewer (RRM) will contact authors in the event of missing data or unclear reporting. Studies will be grouped based on their methodological similarities.

**Table 3:** Variables included in data extraction template

**General Information**	
Study title	
Type of RCT (i.e. standard parallel arm; crossover; cluster)	
Start and end date; duration	
Country	
Funding source	
**Population and setting**	
Gender	
Age group	
Focused diseases/conditions, if any	
Menopause status, if relevant	
Total number of participants	
Source/setting of the population	
Method(s) of recruitment and sampling technique	
**Intervention and comparator**	
Protein source	
Energy content (kcal)	
Protein content (grams)	
Number of participants in each arm	
Compliance	
Dropout rate	
**Additional features of interventions**	
Physical activity	
Supplementary micronutrients	
**Outcomes**	
Muscle health outcomes of interest	
Measurement method	
Unit of analysis	
Time points measured	
Summary statistics at baseline and at all study time points (mean, SD, *P*-value, and 95% CI)	

#### Risk of bias in individual studies

Two reviewers (RRM, SB) will assess the methodological quality and internal validity of eligible trials at the study level using the Cochrane Risk of Bias 2.0 tool (RoB 2) [34]. For each trial that meets eligibility criteria, risk of bias will be assessed across five domains: the randomisation process, deviations from intended interventions, missing outcome data, outcome measurement and the reporting of results. For each domain, the signalling questions listed in the RoB 2.0 will be applied to the individual study, and a risk of bias judgement will be made, either high risk, some concerns or low risk. The overall risk of bias will be determined as follows:

- Overall low risk of bias only if all independent domains are found to have low risk of bias
- Overall high risk of bias if at least one domain presents high risk of bias or if multiple domains raise some concerns
- Overall, some concerns if at least one domain gives this result and no domains give a high risk of bias

Discrepancies will be resolved through discussion between two reviewers (RRM, SB) and a third reviewer if required (CME).

#### Data synthesis

Summary tables will be presented to show key information for each paper including study and participant characteristics, intervention and comparator characteristics, outcomes and RoB 2 category. All studies will be then discussed in a narrative synthesis, and meta-analyses will be performed for each outcome. All analyses will be conducted using RevMan software [Review Manager, version 5.3, 2014].

Where data permits, we will quantify the effect of plant versus animal protein interventions on muscle health in adults by calculating between-group standardised mean difference (SMD) and 95% confidence intervals (95% CI) for each of the muscle outcomes. Results will be presented in a forest plot for each outcome.

Statistical heterogeneity will be assessed using several methods. Each forest plot will be visually assessed for inter-study heterogeneity. The chi-squared test for heterogeneity and the *I*^2^ statistical test will also be conducted, with levels of heterogeneity for the *I*^2^ test defined as follows: low, 0–25%; moderate, 25–50%; high, 75–100% [35]. Later subgroup analyses will be interrogated to explain any heterogeneity found at this stage.

If significant heterogeneity is detected, a meta-analysis will be conducted for each outcome using a random-effects model to account for such inter-study and between-study heterogeneity [36]. Studies with greater than one intervention/plant protein group will be presented as follows: plant protein group 1 vs comparator and associated mean difference and plant protein group 2 vs comparator and associated mean difference. Any studies such as these with > 1 result presented in a meta-analysis will receive a smaller weight in any pooled analysis.

If possible, subgroup analyses will be conducted for the following:

- Male and female populations
- Different life stages, i.e. young adults, midlife and older adults
- Different plant protein sources, i.e. pea protein, soy protein

#### Sensitivity analyses

A sensitivity analysis of studies with a low to medium risk of bias will be undertaken to examine whether studies with a high risk of bias are likely to have affected the result. If possible, another set of analyses separating industry- and nonindustry-funded studies will be undertaken to reveal any potential funding outcome biases.

#### Risk of meta-biases

Several methods will be used to interrogate risk of meta-bias in this review. Funnel plot asymmetry will be interrogated by two reviewers (RRM, SB) who will come to a joint conclusion as to the risk of publication bias in the review. Egger’s test will also be performed to statistically analyse funnel plot asymmetry [37]. However, conclusions drawn regarding publication bias are likely to be tentative based on the small number of studies expected for each separate outcome and thus the limited capacity of these tests to detect publication bias, as well as the potential that Egger’s test has limitations when assessing continuous outcomes [38]. Risk of reporting bias should be limited as any relevant results that are not explicitly reported in studies will be requested from study authors. However, if no response is received from study authors, the risk of reporting bias will be discussed in the review manuscript.

#### Confidence in cumulative evidence

The GRADE framework will be used to assess the certainty of evidence [39, 40]. A separate GRADE assessment will be conducted for each RCT by one reviewer (RRM), and consensus agreement will be sought from the entire review team.

## Discussion

The completed systematic review manuscript is intended to be published in a suitable peer-reviewed journal. Any amendments or deviation from this protocol will be outlined in the later manuscript. Results from this review will be a valuable addition to the area of plant-based and sustainable nutrition, providing a quantitative summary of any muscle health-related trade-offs between plant proteins as a more sustainable protein source, and animal proteins which are of higher quality but less sustainable. With increasing numbers of people adhering to flexitarian, vegetarian and vegan diets, it is necessary to know how plant protein sources compare to traditional animal sources, especially as these populations age and muscle atrophy and disability become a greater concern.

## Data Availability

Data sharing is not applicable to this article as no datasets were generated or analysed at this stage of the review.

## Appendix 1

### Search strategy formatted for use in Cochrane Central Register of Controlled Trials (CENTRAL) database

[mh "dietary proteins"] OR [mh ^"grain proteins"] OR [mh ^"soybean proteins"] OR [mh "dairy products"] OR [mh ^eggs] OR [mh ^"food, fortified"] OR [mh ^"functional food"] OR [mh ^"meat proteins"] OR [mh meat] OR [mh ^nuts] OR [mh "soy foods"] OR [mh "diet, high-protein"] OR [mh ^"diet, vegetarian"] OR [mh ^vegans] OR (diet* NEAR/2 protein*):ti,ab,kw OR (plant NEAR/2 protein*):ti,ab,kw OR (animal NEAR/2 protein*):ti,ab,kw OR (soy NEAR/2 protein*):ti,ab,kw OR (nut NEAR/2 protein*):ti,ab,kw OR (protein NEAR/2 source):ti,ab,kw OR (soy*):ti,ab,kw OR (tofu):ti,ab,kw OR (pea):ti,ab,kw OR (pulses):ti,ab,kw OR (legumes):ti,ab,kw OR (lentil*):ti,ab,kw OR (chickpea*):ti,ab,kw OR (quinoa):ti,ab,kw OR (nut):ti,ab,kw OR (mycoprotein):ti,ab,kw OR (egg):ti,ab,kw OR (dairy):ti,ab,kw OR (milk):ti,ab,kw OR (whey):ti,ab,kw OR (fish):ti,ab,kw OR (poultry):ti,ab,kw OR (chicken):ti,ab,kw OR (meat):ti,ab,kw OR (plant-based):ti,ab,kw OR (vegetarian*):ti,ab,kw OR (vegan*):ti,ab,kw AND ("muscle mass"):ti,ab,kw OR ("fat-free mass"):ti,ab,kw OR (strength):ti,ab,kw OR ("body composition"):ti,ab,kw OR (sarcopenia):ti,ab,kw OR (physical NEAR/2 performance):ti,ab,kw OR (lean NEAR/2 mass):ti,ab,kw OR (muscle NEAR/2 function*):ti,ab,kw OR (physical NEAR/2 function*):ti,ab,kw OR [mh ^"muscle, skeletal"] OR [mh ^"muscle weakness"] OR [mh ^"body composition"] OR [mh ^sarcopenia] OR [mh "muscle strength"] OR [mh ^anthropometry] OR [mh ^"gait analysis"] OR [mh ^gait] OR [mh ^"walking speed"] OR [mh ^"physical functional performance”].

## Appendix 2

### Search strategy formatted for use in Scopus database

( TITLE-ABS-KEY ( "muscle mass" ) OR ( "fat-free mass" ) OR strength OR ( "body composition" ) OR sarcopenia OR ( physical W/2 performance ) OR ( lean W/2 mass ) OR ( muscle W/2 function* ) OR ( physical W/2 function* ) ) AND ( ( TITLE-ABS-KEY ( diet* W/2 protein* ) OR ( plant W/2 protein* ) OR ( animal W/2 protein* ) OR ( soy W/2 protein* ) OR ( nut W/2 protein* ) OR ( protein W/2 source ) ) OR ( TITLE-ABS-KEY ( soy* OR tofu OR pea OR pulses OR legumes OR lentil* OR chickpea* OR quinoa OR nut OR mycoprotein OR egg OR dairy OR milk OR whey OR fish OR poultry OR chicken OR meat OR plant-based OR vegetarian* OR vegan* ) ) ) ) AND ( TITLE-ABS-KEY ( rct ) OR ( "RANDOMI?ED TRIAL" ) OR ( "RANDOMI?ED CONTROLLED TRIAL" ) AND ( LIMIT-TO ( DOCTYPE , "ar" ) ) AND ( LIMIT-TO ( LANGUAGE , "English" ) ) AND LIMIT-TO ( EXACTKEYWORD , "Randomized Controlled Trial” AND ( LIMIT-TO ( SUBJAREA , "MEDI" ) OR LIMIT-TO ( SUBJAREA , "NURS" ) OR LIMIT-TO ( SUBJAREA , "HEAL" ) )

## Appendix 3

Table 4

**Table 4:** Characteristics and key findings of previous systematic reviews which aimed to identify the importance of protein source for muscle health outcomes

Authors, date	Age	Interventions	Outcomes	Key findings
Langer & Carlsohn, 2014 [26]	< 40	Any two protein sources consumed within 90 min of RET	Muscle mass	A combination of whey and casein milk proteins can provide the most perpetual anabolic effects
Messina et al. 2018 [27]	≥ 18	Soy protein vs animal protein (+RET)	Muscle mass Strength (squat and bench press)	Soy and whey protein were found to have similar effects on lean mass accretion and strength (for both squat and bench press)
Lim et al. 2021 [28]	≥ 19	Plant protein vs animal protein (with or without RET)	Muscle mass Strength (squat, bench press, grip strength, peak flexion/extension of leg)	A nonsignificant favourable effect of animal protein on absolute lean mass was seen, and in the subgroup analysis based on age, an anabolic effect was only observed in the younger populations (< 50 years). No statistical difference between plant and animal protein was seen for strength outcomes aside from a favourable effect of animal protein on leg extension in the younger subgroup

## Abbreviations

AWGS: Asian Working Group for Sarcopenia
BIA: Bioelectrical Impedance
CT: Computed tomography
DEXA: Dual-energy X-ray absorptiometry
EAA: Essential amino acids
EWGSOP: European Working Group on Sarcopenia
MD: Mean difference
MRI: Magnetic resonance imaging
PDCAAS: Protein digestibility-corrected amino acid score
RCT: Randomised controlled trial
RoB2: Risk of Bias 2
SMD: Standardised mean difference
SPPB: Short physical performance battery
TUG: Timed Up-and-Go
